# 2-O-Methylhonokiol Suppresses HCV Replication via TRAF6-Mediated NF-kB Activation

**DOI:** 10.3390/ijms22126499

**Published:** 2021-06-17

**Authors:** Suyun Jeong, Young-seok Lee, Kiyoon Kim, Ji-su Yoon, Sungsoo Kim, Joohun Ha, Insug Kang, Wonchae Choe

**Affiliations:** 1 Department of Biomedical Science, Graduate School, Kyung Hee University, Seoul 02447, Korea; suyanjjang@naver.com (S.J.); wetoo123@gmail.com (Y.-s.L.); jichuu94@khu.ac.kr (J.-s.Y.); sgskim@khu.ac.kr (S.K.); hajh@khu.ac.kr (J.H.); iskang@khu.ac.kr (I.K.); 2 Department of Biochemistry and Molecular Biology, School of Medicine, Kyung Hee University, Seoul 02447, Korea; soowonsky@gmail.com

**Keywords:** 2-O-methylhonokiol, hepatitis C virus, nuclear factor kappa-light-chain-enhancer of activated B cells, innate immune response

## Abstract

Hepatitis C virus (HCV) is associated with various liver diseases. Chronic HCV infection is characterized by an abnormal host immune response. Therefore, it is speculated that to suppress HCV, a well-regulated host immune response is necessary. 2-O-methylhonokiol was identified by the screening of anti-HCV compounds using *Renilla* luciferase assay in Huh 7.5/Con 1 genotype 1b replicon cells. Here, we investigated the mechanism by which 2-O-methylhonokiol treatment inhibits HCV replication using real-time PCR. Our data shows that treatment with 2-O-methylhonokiol activated innate immune responses via nuclear factor kappa-light-chain-enhancer of activated B cells (NF-kB) pathway. Additionally, the immunoprecipitation result shows that treatment with 2-O-methylhonokiol augmented tumor necrosis factor receptor (TNFR)-associated factor 6 (TRAF6) by preventing p62 from binding to TRAF6, resulting in reduced autophagy caused by HCV. Finally, we reproduced our data with the conditioned media from 2-O-methylhonokiol-treated cells. These findings strongly suggest that 2-O-methylhonokiol enhances the host immune response and suppresses HCV replication via TRAF6-mediated NF-kB activation.

## 1. Introduction

Hepatitis C virus (HCV) infection causes chronic liver diseases such as liver cirrhosis and liver cancer [1]. There are a total of seven genotypes of HCV [2], which include separate genotypes that are easily infected by race [3]. In particular, HCV genotype 1 has the highest incidence in the world of 46.2% [4]. Of the 53% of genotype 1 cases for which the subtype was specified, 99% were subtypes 1a and 1b ((31% and 68%), respectively) [4]. Currently, there is no available HCV vaccine, but the treatment of chronic HCV has made rapid progress. The existing standard treatment, pegylated-interferon (IFN) α + ribavirin combined therapy, has been reduced in utility due to severe side effects, along with relatively low therapeutic effects [5]. In recent years, direct acting antiviral (DAA) therapy has been used to suppress HCV replication by acting directly on certain areas of HCV non-structural proteins. Usually, two types of DAA with different modes of action are administered at the same time [6] or additional ribavirin are administered to patients who are difficult to treat. In addition, the rate of treatment caused by pangenotypic DAAs (glecaprevir/pibrentasvir) is over 98% [7]. However, for patients which cirrhosis or re-treatment, this treatment is highly likely to fail due to reinfection. Additionally, for young ages, pangenotypic DAA treatment faces high limitations [8]. Additionally, the cost of this treatment is high, and drug tolerance and persistent HCC risks after cure are present [5]. Therefore, many studies continue to address these issues [9].

Honokiol is a lignan isolated from the bark of the trees belonging to the genus, *Magnolia* [10]. Along with magnolol, 4-O-methylhonokiol, and obovatol, it has been identified as one of the chemical compounds in traditional herbal medicine. Herbal medicine has the advantage of being very cheap and having fewer side effects compared to conventional medication [11]. Recently, honokiol has been shown to inhibit HCV infection in vitro by targeting cell entry and replication. However, the mechanism by which honokiol inhibits HCV replication remains to be elucidated [12]. In addition, several honokiol derivatives have been identified, but their functional roles have not yet been explored, especially in HCV replication.

The human immune system develops in two ways: the innate immunity to immediately protect the spread and movement of foreign pathogens throughout the body; and the adaptive immunity, which takes several days to become protective, and remove a specific antigen [13]. Innate immune is a process that occurs immediately after infection, which directly forms proinflammatory cytokine and regulates adaptive immunity. Innate immunity is developed when pattern recognition receptors (PRRs), including retinoic acid-inducible gene I product (RIG-I)-like receptors (RLRs) and Toll-like receptors (TLRs), recognizes the pathogen-associated molecular patterns (PAMPs) of pathogens [14]. Two PRRs, RLRs, and TLRs are considered important to activate antiviral responses in an organism infected with HCV. Both recognize double-stranded RNA, essential in the HCV replication, and induce the synthesis of IFNs. RLRs signaling pathway activates NF-kB, the transcription factor for the expression of the inflammatory genes [15]. However, HCV has a mechanism that avoids the innate immune response of the host to keep the virus in the host cells [16]. For example, autophagy occurs within host cells to maintain HCV and suppress the host innate immune response by depleting the TRAF6, which is involved in the formation of proinflammatory cytokines [17]. In particular, NF-kB has been known as an important regulator for the inducible gene expression in the host immune system [18]. HCV infection inhibits TNF-α-induced NF-kB activity [19]. In particular, HCV NS3 protein interacts with linear ubiquitin chain assembly complex (LUBAC) to suppress NF-kB activation [20]. Therefore, Increasing the host’s immunity against HCV infection may be a good strategy.

Therefore, in this study, we investigated the mechanism of inhibitory effect of 2-O-methylhonokiol, a derivative of honokiol, in HCV replication, using Huh 7.5/Con 1 genotype 1b replicon cells. The aim of our study is to gain insight into HCV RNA replication mechanisms through induction of NF-kB, which is related to host innate immune response. 

## 2. Results

### 2.1. Inhibitory Effects of 2-O-Methylhonokiol on HCV Replication

**Figure 1:**
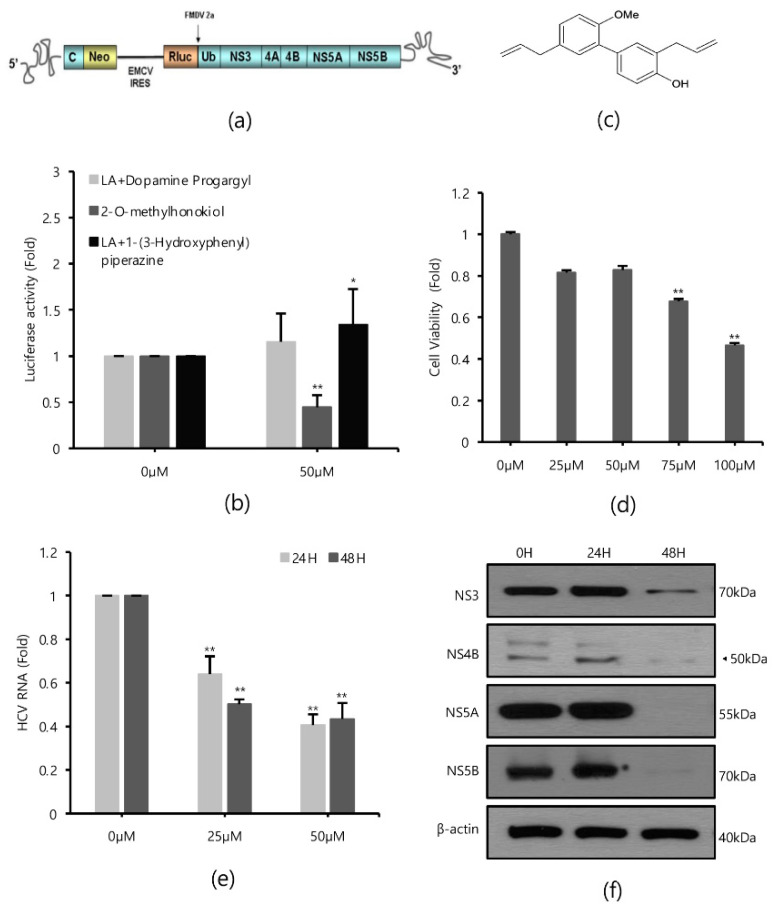
Effects of 2-O-methylhonokiol on HCV replication. (**a**) The structure of Huh7.5/Con1 replicon. (**b**) Huh7.5/Con1 cells were treated with three different promising compounds of 50 μM each for 24 h. HCV replication was measured by *Renilla* luciferase assays. (**c**) The chemical structure of 2-O-methylhonokiol. (**d**) Huh7.5/Con1 cells were treated with the indicated concentrations of 2-O-methylhonokiol for 24 h. Cell viability was measured by MTS assay. (**e**) After treatment with 25 or 50 μM of 2-O-methylhonokiol for 24 or 48 h, HCV RNA levels were measured by qRT-PCR. (**f**) Western blotting analysis of HCV non-structural protein (NS3, NS4B, NS4A, and NS5B) using Huh7.5/Con1 cells treated with 50 μM 2-O-methylhonokiol for (24 or 48) h. In each experiment, 0 H or 0 μM means a control. The values are shown as the mean ± SD of three independent experiments. * *p* < 0.05, ** *p* <0.01 versus untreated control.

*Renilla* luciferase assays were performed in Huh 7.5/Con 1 genotype 1b replicon cells in the absence or presence of 80 compounds with antioxidant properties to identify inhibitors of HCV replication, since HCV genotype 1b had the highest incidence (Figure 1a). As a result of repeated quantitative analysis of HCV replication, it was confirmed that 2-O-methylhonokiol inhibited HCV replication by more than 50% relative to that in equal volume DMSO-treated cells (Figure 1b). Figure 1c shows the structure of 2-O-methylhonokiol. The cytotoxicity and anti-HCV efficacy of 2-O-methylhonokiol were evaluated. As a result of MTS analysis, 2-O-mehtylhonokiol did not significantly affect cell viability up to a concentration of 50 μM for 24 h (Figure 1d). However, Huh 7.5/Con 1 genotype 1b replicon at a concentration of 50 μM was suppressed by more than 50%, compared with that in equal volume DMSO-treated-cells (Figure 1e). Therefore, the following experiments were performed with 50 μM 2-O-methylhonokiol. To confirm the inhibitory effect of 2-O-methylhonokiol on HCV replication, expressions of HCV non-structural proteins, namely NS3, NS4B, NS5A, and NS5B, were analyzed by Western blot assay. As expected, treatment with 2-O-methylhonokiol significantly suppressed the expression of viral non-structural proteins, especially at 48 h, while there was no significant change in protein expression at 24 h (Figure 1f).

### 2.2. Comparison of 2-O-Methylhonokiol and Honokiol on Cytotoxicity and HCV Replication Inhibition

**Figure 2:**
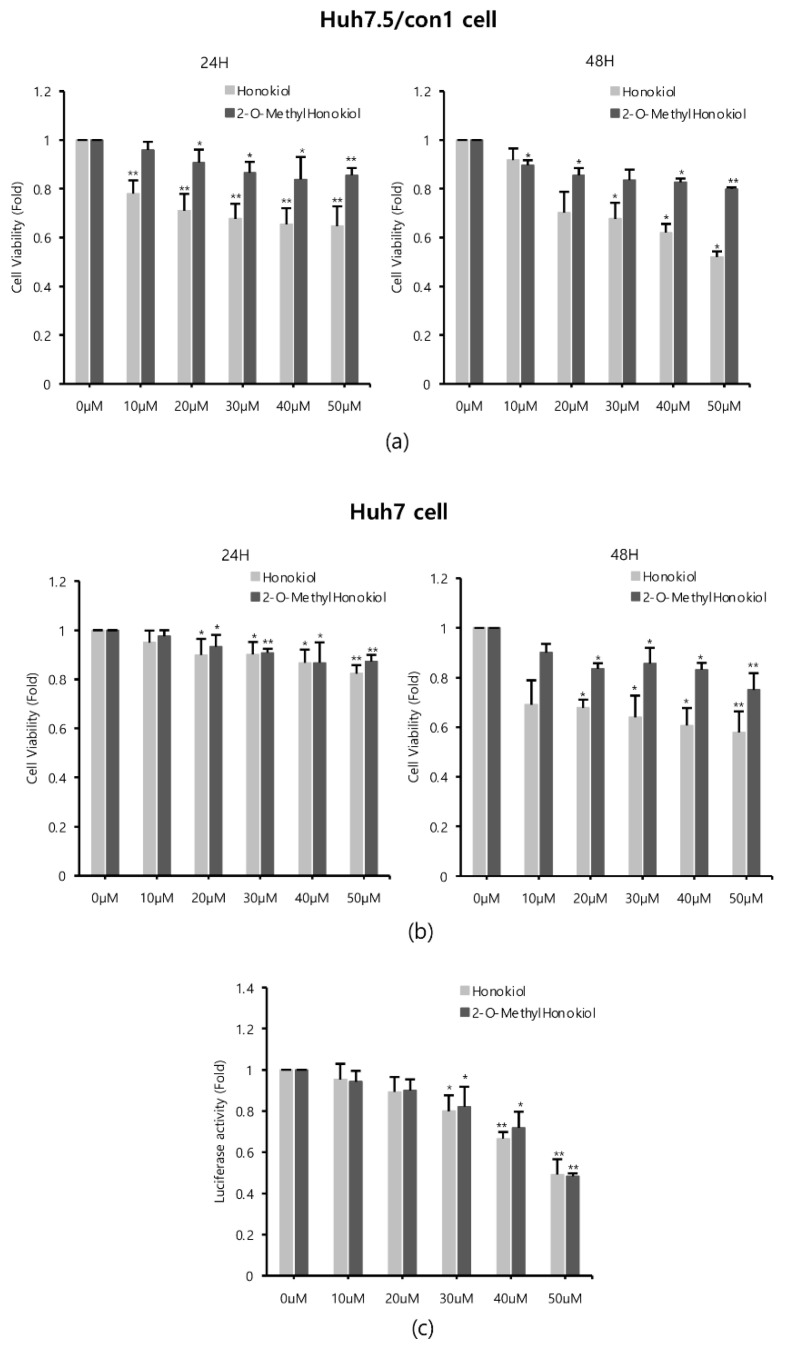
Comparison of 2-O-methylhonokiol and honokiol on cytotoxicity and HCV replication inhibition. (**a**) Huh7.5/Con1 cells and (**b**) Huh7 cells were treated with honokiol and 2-O-mehtylhonokiol at the indicated doses for 24 or 48 h. Cell viability was measured by MTS assay. (**c**) After Huh7.5/Con1 cells were treated with honokiol or 2-O-methylhonokiol at the indicated doses for 24 h, the levels of HCV RNA were measured by *Renilla* luciferase assay. In each experiment, 0 μM means a control. The values are shown as the mean ± SD of three independent experiments. * *p* < 0.05, ** *p* <0.01 versus untreated control.

Since honokiol was already reported to inhibit HCV replication [12], we compared the effects of honokiol and 2-O-methylhonokiol on cytotoxicity and anti-HCV activity. Cell viability was measured by MTS assay, after Huh7.5/Con1 cells and Huh7 cells were treated with various concentrations of honokiol and 2-O-methylhonokiol for 24 or 48 h. When Huh7.5/con1 cells were treated with 50 μM for 24 h, 2-O-mehtylhonokiol treatment showed about 20% higher cell viability compared to honokiol treatment. At 48 h treatment, 2-O-methylhonokiol treatment showed about 40% higher cell viability compared to honokiol treatment. Therefore, In Huh7.5/con1 cells, 2-O-methylhonokiol was less toxic than honokiol (Figure 2a). On the other hand, there was no significant change in cell viability in Huh7 cells treated for 24 h. However, in Huh7 cells treated with 50 μM for 48 h, 2-O-methylhonokiol was less toxic by approximately 20% than honokiol (Figure 2b). Next, we analyzed HCV replication with *Renilla* luciferase assay to compare the difference of anti-HCV activity of two compounds. There was little difference in the ability of the honokiol and 2-O-methylhonokiol to suppress HCV replication (Figure 2c). Thus, we concluded that 2-O-methylhonokiol is a more promising candidate than honokiol as an anti-HCV drug.

### 2.3. 2-O-Methylhonokiol Suppresses HCV Replication through NF-kB Activation

Next, we investigated how 2-O-methylhonokiol affects NF-kB activity and the host innate immune system, and compared with honokiol. First, we monitored whether honokiol and 2-O-methylhonokiol activates NF-kB by qRT-PCR and immunoblotting analysis.

**Figure 3:**
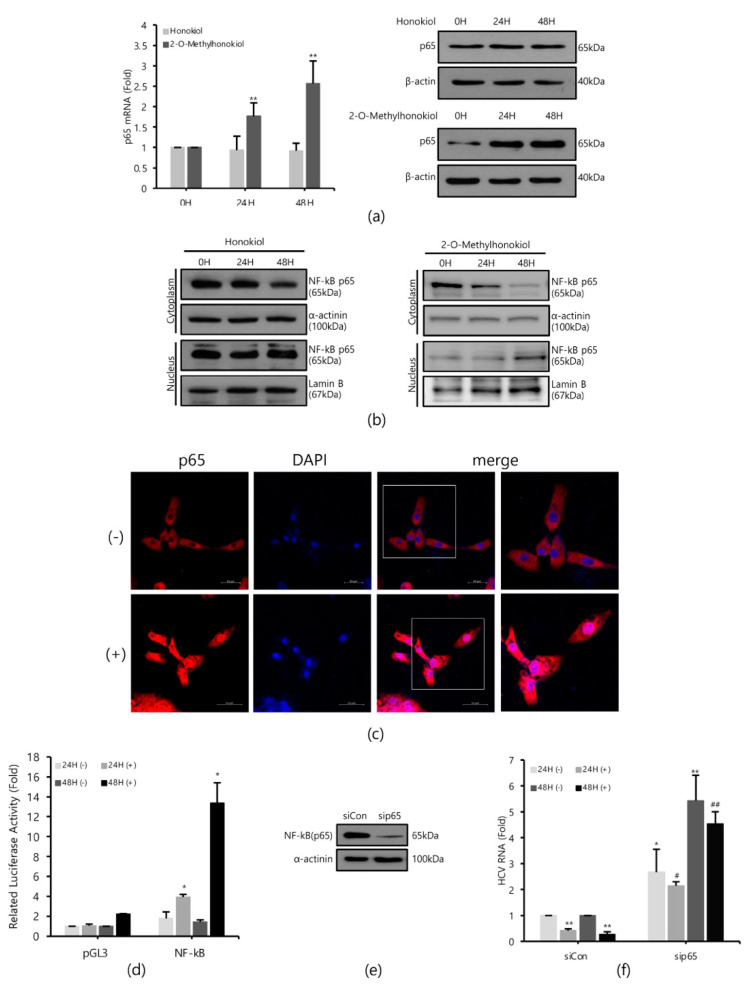
2-O-methylhonokiol inhibits HCV replication via NF-kB activation, but honokiol does not. (**a**) After treatment of 50 μM of 2-O-methylhonokiol and honokiol for 24 or 48 h, p65 mRNA levels were measured by qRT-PCR (left), and Western blotting analysis of p65 protein (right), using Huh7.5/Con1 cells. (**b**) Cytosol or nuclear fractions were isolated and analyzed by Western blotting in huh7.5/Con1 cells treated with 50 μM 2-O-methylhonokiol (right), and honokiol (left), for 24 or 48 h. Lamin B (nuclear marker) and α-actinin (cytosolic marker) immunoblotting were also included to confirm the purity of the nuclear and cytosolic fractions, respectively. (**c**) Huh7.5/Con1 cells were treated with or without 50 μM 2-O-methylhonokiol for 24 h, and then subjected to immunofluorescence with anti-p65 antibody (red). DAPI (blue) was used for nuclear staining. Scale bar = 50 μm. (**d**) Huh7.5/Con1 cells were transfected with pGL3-Luc-NF-kB plasmid or the empty pGL3 vector for 24 h. Luciferase activity was measured after treatment with or without 50 μM 2-O-methylHonokiol for 24 or 48 h. Luciferase activity was expressed relative to that in DMSO-treated cells. (**e**) Expression of NF-kB(p65) protein in control (si-Con) and NF-kB(p65)-interfered (si-RelA/p65) Huh7.5/Con1 cells was shown. (**f**) After transfection with si-Con or si-RelA/p65 for 48 h, Huh7.5/Con1 cells were treated with or without 50 μM 2-O-methylhonokiol for 24 or 48 h. The HCV RNA level were then analyzed by qRT-PCR. The values are shown as the mean ± SD of five independent experiments. * *p* < 0.05, ** *p* < 0.01 versus untreated control or versus the untreated siCon-transfected control; # *p* < 0.05, ## *p*< 0.01 versus the 2-O-methylhonokiol-treated, siCon-transfected control; one-way ANOVA with Tukey’s multiple comparisons test.

Figure 3a shows that while 2-O-mehtylhonokiol treatment effectively increased the RelA/p65 mRNA and protein levels by about (2–3)-fold, honokiol treatment did not change the RelA/p65 mRNA and protein levels. While 2-O-methylhonokiol treatment effectively increased the nuclear RelA/p65 pool, which indicates nuclear translocation of RelA/p65 (Figure 3b, right; and Figure 3c), honokiol did not increase the nuclear RelA/p65 pool (Figure 3b, left). Therefore, we found that the difference between honokiol and 2-O-methylhonokiol in inhibiting HCV replication is whether they cause NF-kB activation. 

To confirm the nuclear translocation of RelA/p65 subunit of NF-kB, we used NF-kB-luciferase reporter assay in Huh7.5/Con1 cells after the 2-O-methylhonokiol treatment. When treated with 2-O-methylhonokiol for 48 h, we observed almost 15-folds increase in NF-kB-luciferase activity, compared to equal volume DMSO-treated cells (Figure 3d).

To further analyze the role of NF-kB activation in the inhibitory effect of 2-O-methylhonokiol in HCV replication, RelA/p65 knockdown by siRNA experiments was utilized. Huh 7.5/Con 1 genotype 1b replicon cells were transfected with si-RelA/p65 for 48 h, and RelA/p65 knockdown efficiency was verified by Western blotting (Figure 3e). Then HCV RNA replication was analyzed in RelA/p65 knockdown for 48 h, after treatment with 2-O-methylhonokiol for 24 or 48 h. Figure 3f shows that, as expected, 2-O-methylhonokiol significantly suppressed HCV replication in si-Con. However, RelA/p65 knockdown abolished the suppressive effects of 2-O-methylhonokiol in HCV replication. Interestingly, compared to siCon, HCV replication in NF-kb/p65-knockdown cells were increased by about (2–5)-fold, independently of 2-O-methylhonokiol. This was highlighted by the difference between HCV RNA levels in siCon/2-methylhonokiol-treated versus si-RelA/p65/2-mehtylhonokiol-treated HCV cells, suggesting that NF-kB(p65) mediated HCV replication. So, our data suggest that 2-O-methylhonokiol inhibits HCV replication through NF-kB activation.

### 2.4. 2-O-Methylhonokiol Enhances TRAF6-Mediated NF-kB Activation

**Figure 4:**
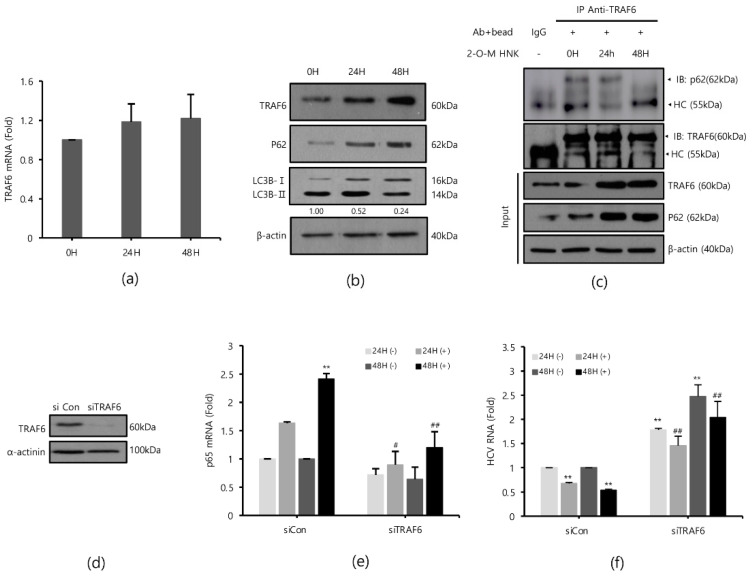
2-O-methylhonokiol enhances TFAF6-mediated NF-kB activation. After treatment with 50 µM 2-O-methylhonokiol for 24 or 48 h, (**a**) TRAF6 mRNA levels were analyzed by qRT-PCR, and (**b**) the protein levels of TRAF6, p62, and LC3B were analyzed by Western blotting. (**c**) Immunoprecipitation was performed with Huh7.5-Con1 cells with or without 50 μM 2-O-methylhonokiol for 24 or 48 h using antibodies against TRAF6, and precipitated protein complexes were probed with anti-p62 antibodies. The total cell lysates were also used for immunoblotting to serve as the input control. HC, antibody heavy chain; 2-O-M HNK, 2-O-methylhonokiol. (**d**) Expression of TRAF6 protein in control (si-Con) and TRAF6-interfered (si-TRAF6) Huh7.5/Con1 cells is shown. After transfection with si-Con or si-TRAF6 for 48 h, Huh7.5/Con cells were treated with or without 50 μM 2-O-methylhonokiol for 24 or 48 h. (**e**) The mRNA level of p65 and (**f**) the HCV RNA level were then analyzed by qRT-PCR. The mRNA level of p65 and HCV RNA level were normalized against β-actin RNA. In each experiment, ‘0H’ means a DMSO control. The values are shown as the mean ± SD of five independent experiments. ** *p* < 0.01 versus untreated control or versus the untreated siCon-transfected control; # *p* < 0.05, ## *p*< 0.01 versus the 2-O-methylhonokiol-treated, siCon- transfected control or versus CQ or 2-O-M HNK; one-way ANOVA with Tukey’s multiple comparisons test.

TRAF6 is an important signaling molecule that mediates the activation of NF-kB and is depleted by HCV [17]. Therefore, we hypothesized that 2-O-methylhonokiol may reactivate TRAF6-mediated NF-kB signaling pathway. First, we monitored the expression levels of TRAF6 mRNA and protein level after treatment with 2-O-methylhonokiol for 24 or 48 h. Figure 4a shows that the TRAF6 mRNA levels were not significantly affected by treatment with 2-O-methylhonokiol, compared to equal volume DMSO-treated cells; however, treatment with 2-O-methylhonokiol significantly increased the expression levels of TRAF6 protein levels, compared to equal volume DMSO-treated cells (Figure 4b).

To confirm our data, we measured the level of p65 mRNA expression and the level of HCV replication in TRAF6 knockdown. TRAF6 knockdown was verified by Western blotting (Figure 4d). As expected, treatment with 2-O-methylhonokiol activated the transcription of RelA/p65 by 2 folds in si-Con. However, the activated RelA/p65 transcription was abolished in TRAF6 knockdown (Figure 4e). Additionally, we confirmed that the increased levels of NF-kB(p65) mRNA by 2-O-methylhonokiol are more likely due to an increase in transcription itself, rather than mRNA stability (Figure S1 of the Supplementary Information (SI)). Figure 4f shows that the suppressed HCV replication by 2-O-methylhonokiol was reactivated by (2–3)-fold in TRAF6 knockdown. Interestingly, compared to siCon, HCV RNA replication was increased independently of 2-O-methylhonokiol by about (1.5–2)-fold in TRAF6-knockdown cells. Overall, our results suggested that 2-O-methylhonokiol inhibits HCV RNA replication through activation of TRAF6-mediated NF-kB pathway.

Several studies have shown that HCV replication induces LC3II expression and reduces the expression of p62/SQSTM1, an autophagy adapter that interacts with TRAF6 and LC3, leading to autophagy [21,22,23,24,25]. Therefore, we monitored whether treatment with 2-O-methylhonokiol suppresses autophagy by Western blotting. Figure 4b shows that p62/SQSTM1 was induced by treatment with 2-O-methylhonokiol. Additionally, the LC3II/LC3I ratio decreased, suggesting that treatment with 2-O-methylhonokiol diminishes autophagy. p62 has been reported to bind to TRAF6 in HCV-infected cells to proceed with autophagic degradation of TRAF6 [17]. Immunoprecipitation was performed to investigate the role of 2-O-methylhonokiol in p62-TRAF6 interaction. We found that the physical binding of p62 and TRAF6 was abolished by 2-O-methylhonokiol treatment, especially at 48 h, suggesting that treatment with 2-O-methylhonokiol may decrease autophagy (Figure 4c). Our data showed that treatment with 2-O-methylhonokiol increased TRAF6 by preventing p62 from binding to TRAF6. Then, we tested the effect of 2-O-mehtylhonokiol on viral replication in the presence of autophagy inhibitors. Additionally, we showed that combination treatment with 2-O-methylhonokiol and chloroquine, a well-known autophagy inhibitor [26], reduced HCV replication more than each treatment, suggesting that 2-O-methylhonokiol displays a synergistic effect with chloroquine (Figures S1 and S2). Finally, 2-O-methylhonokiol directly affected p62 and LC3, inhibiting autophagy that occurs during HCV replication. We also found that HCV replication was inhibited by activation of the TRAF6-mediated NF-kB by inhibiting depletion of the TRAF6, which became autophagic degradation by p62 in HCV replication. 

### 2.5. 2-O-Methylhonokiol Stimulates Innate Immune Responses

**Figure 5:**
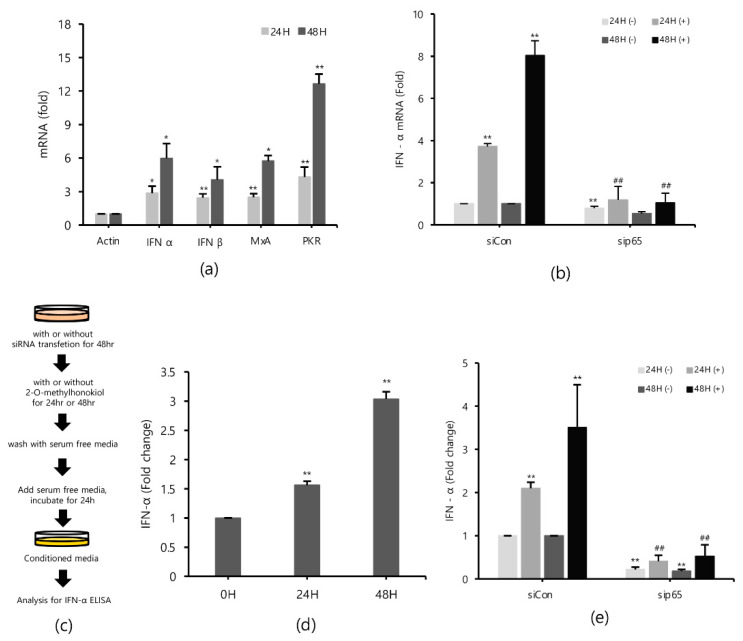
2-O-methylhonokiol activates innate immune response. (**a**) The mRNA levels of IFN-α, IFN-β, MxA, and PKR in Huh7.5/Con1 cells treated with 50 μM 2-O-methylhonokiol for 24 or 48 h were analyzed by qRT-PCR. After transfection with si-Con or si-RelA/p65 for 48 h, Huh7.5/Con1 cells were treated with or without 50 μM 2-O-methylhonokiol for (24 or 48) h. (**b**) The mRNA level of IFN-α in treated cells was confirmed through qRT-PCR, and (**e**) the amount of IFN-α secretion was confirmed through ELISA kits. (**c**) Schematic of the ELISA process. (**d**) huh7.5/Con 1 cells were treated with 50 µM 2-O-methylhonokiol for 24 or 48 h. After incubation in serum-free DMEM for 24 h, the supernatants were harvested, and analyzed using IFN-α ELISA kits. The values are shown as the mean ± SD of five independent experiments. * *p* < 0.05, ** *p* < 0.01 versus untreated control or versus the untreated siCon-transfected control; ## *p*< 0.01 versus the 2-O-methylhonokiol-treated, siCon-transfected control; one-way ANOVA with Tukey’s multiple comparisons test.

Type I interferons (IFNs) are a family of primitive cytokines that respond to infection with various pathogens, such as HCV [27,28]. HCV replication has been reported to be inhibited when IFN-α is upregulated [29]. Expressions of innate immune response-related genes, such as protein kinase R (PKR) [30] and myxovirus resistance protein 1 (MxA) [31,32] induced by IFNs, also suppress HCV replication. To identify the effects of 2-O-methylhonokiol on IFNs and IFNs down-stream effectors in inhibition of HCV replication, mRNA expressions of IFN-α, IFN-β, MxA, and PKR genes were measured by qRT-PCR. Figure 5a shows that 2-O-methylhonokiol upregulated mRNA expressions of IFN-α, IFN-β, MxA, and PKR genes, compared with non-treated, especially for 48 h. Consistently, 2-O-methylhonokiol treatment induced IFN-α secretion 2–3 times, compared to no treatment (Figure 5d). In order to confirm the results, we measured the expression of IFN-α by qRT-PCR or IFN-α ELISA in RelA/p65 knockdown. Figure 5b shows the qRT-PCR analysis that revealed that the upregulated mRNA expression of IFN-α was abolished in RelA/p65 knockdown. Induced secretion of IFN-α also was also abolished in RelA/p65 knockdown (Figure 5e). Altogether, our results suggest that treatment with 2-O-methylhonokiol inhibits HCV replication by activating innate immune responses via the NF-kB pathway.

### 2.6. Conditioned Media from 2-O-Methylhonokiol-Trated Cells also Inhibit HCV Replication

**Figure 6:**
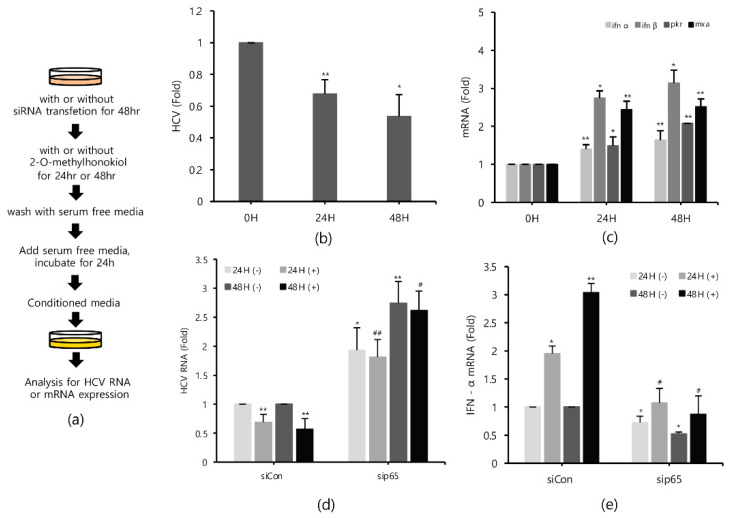
Conditioned media derived from 2-O-methylhonokiol-treated cells also suppresses HCV replication and activates innate immune responses. (**a**) Schematic of the experimental process. The conditioned medium cells were collected from the cell lines treated with 50 μM 2-O-methylhonokiol. The levels of (**b**) HCV RNA, and (**c**) the indicated mRNAs, were measured by qRT-PCR, using the conditioned media-treated cell lines. After transfection with si-Con or si-RelA/p65 for 48 h, Huh7.5/Con1 cells were treated with or without 50 μM 2-O-methylhonokiol for 24 or 48 h. The levels of (**d**) HCV RNA, and (**e**) IFN-α mRNA, were then analyzed by qRT-PCR, after treatment with 50 μM 2-O-methylhonokiol for (24 or 48) h. The values are shown as the mean ± SD of three independent experiments. * *p* < 0.05, ** *p* <0.01 versus untreated control, or versus the untreated siCon-transfected control; # *p* < 0.05, ## *p*< 0.01 versus the 2-O-methylhonokiol-treated, siCon-transfected control; one-way ANOVA with Tukey’s multiple comparisons test.

**Figure 7:**
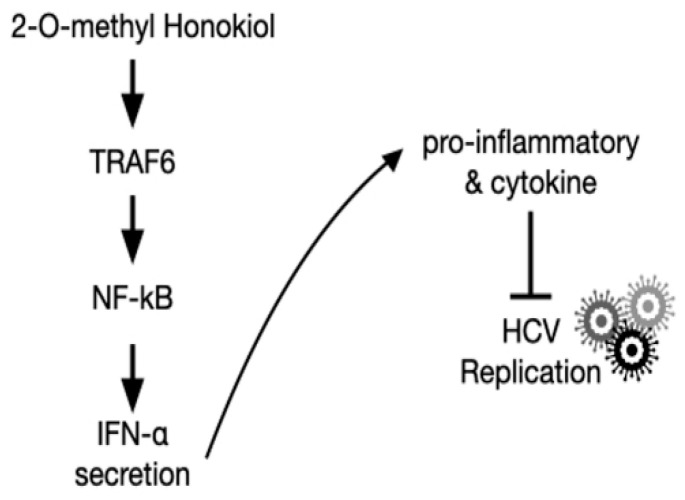
Schematic of the mechanism by which 2-O-methylhonokiol suppresses HCV replication.

Finally, in order to confirm that 2-O-methylhonokiol stimulates innate immune responses to inhibit HCV replication, we collected the supernatants in the conditioned medium from Huh7.5/Con1 cells and examined their effects on HCV replication. Compared to cells treated with the equal volume of DMSO, conditioned media from 2-O-methylhonokiol-treated cells significantly inhibited HCV replication by 50% and increased mRNA levels of IFN-α, IFN-β, PKR, and MxA by about (2–3)-fold (Figure 6b,c). Additionally, we used RelA/p65-knockdown cells to confirm that 2-O-methylhonokiol activates NF-kB signal to inhibit HCV replication. Figure 6d shows that treatment with the conditioned media significantly reduced HCV replication in si-Control. However, HCV replication was increased in RelA/p65 knockdown. Moreover, we could not find any transcriptional upregulation of IFN-α in p65 knockdown, especially for 48 h (Figure 6e). All these together, we concluded that treatment with 2-O-methylhonokiol inhibits HCV replication by activating innate immune responses via the NF-kB pathway (Figure 7).

## 3. Discussion

In this paper, we demonstrated that 2-O-methylhonokiol suppresses HCV replication through NF-kB activation and induces host innate immune responses. Honokiol has been reported to have the effect of inhibiting HCV replication, although the mechanism is still unclear [12].

The compound schematic in Figure 1c shows that the 2-O-methylhonokiol is one of honokiol derivatives. Honokiol is an herbal medicine derived from several species of magnolia native to many parts of the world. Medicinal plants have proven that they can be used to cope with many diseases, including cancer and viral diseases [33]. Herbal medicines are easily accessible to the public in terms of price, and are much safer than modern synthetic drugs [11]. Furthermore, herbal medicine still holds an important position in the modern pharmaceutical industry, due to its small side effects, and synergies from combinations of compounds [34]. Although herbal medicine is still less common in the United States and Europe, much more research is being conducted since scientific evidence of the effectiveness has become more widely available [35]. However, there are still shortcomings, such as the lack of drug effects due to low in-body absorption rates, and the complexity of drug discovery, such as the inability to purify individual ingredients on a sufficient scale [33]. 2-O-methylhonokiol has less cytotoxicity than honokiol, while 2-O-methylhonokiol exhibits almost the same inhibitory effects as honokiol in HCV replication (Figure 2). Therefore, our data suggest that as anti-HCV drug, 2-O-methylhonokiol may be a more beneficial treatment than honokiol.

Interestingly, we showed that 2-O-methylhonokiol treatment activated NF-kB activation for its antiviral activity in Huh 7.5/Con 1 genotype 1b replicon, and confirmed it using RelA/p65 knockdown cells (Figure 3). However, NF-kB pathway was not altered in honokiol-mediated inhibition of HCV replication. This suggested that the methyl group of 2-O-methylhonokiol may play an important role in NF-kB activation, although further studies are needed to differentiate the inhibitory mechanisms of honokiol and 2-O-methylhonokiol in HCV replication. NF-kB pathway was originally reported as a host factor supporting HCV subgenomic replicon replication in Huh 7.5 cells [36]. However, it was recently reported that NF-kB inactivation resulted in the enhanced HCV replication of Huh 7.5/Con1b replicon [37] and HCV1a infected Huh7.5.1 cells [38]. While there are few reports about 2-O-methylhonokiol, various results have recently been published on the regulation of NF-kB by honokiol. Honokiol was reported to suppress NF-kB activated by carcinogens and inflammatory stimuli through the suppression of Akt and activation of IKK in many cell lines [39]. In contrast, it was reported that honokiol treatment did not alter NF-kB pathway in dengue virus infected Huh7 cells [40]. Moreover, honokiol was also reported to efficiently motivate the NF-kB pathway by 17-fold for antiviral activity in grass carp reovirus infected-*C. idella* kidney cells [41]. Therefore, there has been controversy over the role of honokiol in the NF-kB pathway. The reason for this discrepancy is unknown, but it may be attributable to difference in cell types and infected viruses.

In addition, TRAF6 is an important mediator for the activation of NF-kB, leading to the expression of antiviral cytokines [17,42,43,44,45]. Figure 4b shows that treatment with 2-O-methylhonokiol increased TRAF6 proteins by preventing p62 from binding to TRAF6. We showed that TRAF6 mediated the mechanism by which 2-O-methylhonokiol activates NF-kB pathway (Figure 4e). Moreover, HCV replication was increased in TRAF6 knockdown, suggesting that TRAF6 may be directly related to HCV replications (Figure 4f).

Autophagy is known to eliminate the infection of bacterial pathogens [46] and virus [47]. However, HCV-induced autophagy increases HCV replication, rather than inhibiting it [48]. In HCV-induced autophagy, it was reported that p62, which is important for selective autophagy, increases HCV replication by degrading TRAF6 [17]. Figure 4b shows that 2-O-methylhonokiol treatment prevented the degradation of p62, and reduced LC3II/LC3I ratio to inhibit HCV-induced autophagy.

In addition, we showed that 2-O-methylhonokiol upregulated innate immune response genes, such as IFNs, MxA, and PKR (Figure 5). Finally, we confirmed our data using conditioned media from 2-O-methylhonokiol-treated cell lines (Figure 6). Our findings suggest that 2-O-methylhonokiol might be a promising candidate to enhance host innate immunity against HCV through NF-kB pathway.

## 4. Materials and Methods 

### 4.1. Cells and Compounds

Huh7.5 cells expressing the HCV genotype 1b subgenomic replicon (Con1/SG-Neo(I)hRluc2aUb) were cultured in Dulbecco’s modified Eagle’s medium (DMEM) with 10% fetal bovine serum, 100 U/mL penicillin-streptomycin, 2 mM non-essential amino acid (Gibco, Grand Island, NY, USA), and 0.3 mg/mL geneticin (Thermo Fisher Scientific, Waltham, MA, USA) [29]. Huh7 cells were cultured in RPMI1640 supplemented with 10% FBS. All cells were cultured in a humidified atmosphere (5% CO_2_, 37 °C). The antioxidant compounds, including 2-O-methylhonokiol, were kindly gifted by Dr. Jongho Park, Han-bat University, Daejeon, Korea. Chloroquine was purchased from Sigma-Aldrich (catalog no. C6628-25G; Sigma-Aldrich, St. Louis, MO, USA). 2-O-methylhonokiol and chloroquine were dissolved in dimethylsulfoxide (DMSO). Stock solutions of honokiol, 2-O-methylhonokiol, and chloroquine (final concentration, 50 mM) was prepared in DMSO, stored at −20 °C, and diluted with fresh complete medium immediately before use. An equal volume of DMSO (final concentration <0.1%) was always used as a control.

### 4.2. Plasmids, siRNAs Transfection

X-treamgene HP DNA transfection reagents were used according to the manufacturer’s instruction (catalog no. 06 366 546 001; Roche, Swiss). The following siRNA were purchased from Dharmarcon (Dharmacon, Denver, CO, USA): siGenome human RelA (D-003533-04-0010), siGenome human TRAF6 (D-004712-04-0020), and control siRNA (D-001210-02-20). We transfected with each siRNA(100 nM) for 48 h using Viromer BLUE transfection reagent (catalog no. VB-01LB-00; lipocalyx, Germany), according to the manufacturer’s instructions.

### 4.3. Cell Viability Assay

Cells were seeded in 24-well plate. After 24 h, the cells were incubated with varying concentrations of the indicated compounds for 24 or 48 h. After each time treatment, Cell Viability was measured by using Chromo-CK^™^ Cell Viability Assay (MTS) reagent (monobio, Seoul, Korea; catalog no. CH-10000), which was added to the medium for 1 h at 37 °C. Absorbance was measured with Microplate Reader (Molecular Device, Sunnyvale, CA, USA) at 450 nm. 

### 4.4. Luciferase Reporter Assay

*Renilla* luciferase assay: Briefly, Huh7.5/Con1 Cells were plated at 1 × 10^5^ per well in 24-well plates. After 24 h, the cells were treated the indicated compounds for 24 h. After treatment, the cells were washed with 1X PBS. Then 200 μL of 1X *Renilla* luciferase assay lysis buffer was added to each culture well, and the culture plates were placed on orbital shaker with gentle shaking for 15 min at room temperature. The cell lysate was transferred to a 1.5 mL microcentrifuge tube. Cell lysate was obtained by centrifugation at top speed for 30 s in a refrigerated microcentrifuge. The *Renilla* luciferase activity was determined using the *Renilla* luciferase assay kit (catalog no. E2810; Promega, Madison, WI, USA), according to the manufacturer’s instructions. Luminescence was measured with the microplate reader (Molecular Device, Sunnyvale, CA, USA).

Luciferase assay: Huh7.5/Con1 Cells were plated at 1 × 10^5^ per well in 24-well plates. The next day, the cells were transiently transfected with the empty pGL-3 vector (4 µg) or Luc-NF-kB plasmid (4 µg) using Lipofectamine^™^ 3000 reagent for 24 h. All plasmids were kindly gifted by Dr. Joohun Ha (Kyung Hee University, Seoul, Korea). Following transfection, the transfected cells were treated with 2-O-methylhonokiol for 24 or 48 h. The NF-kB-dependent luciferase activity was normalized to the protein concentration measured by Bio-Rad protein assay. Luminescence was measured with the microplate reader (Molecular Device, Sunnyvale, CA, USA).

### 4.5. Quantitative Real-Time Reverse Transcription Polymerase Chain Reaction (qRT-PCR)

Total RNA was isolated from Huh7.5 cells expressing the HCV genotype 1b subgenomic replicon by using a Trizol reagent (catalog no. 15596026; Invitrogen, Waltham, MA, USA). A cDNA was synthesized from 1.0 µg total RNA using RevertAid First Strand cDNA Synthesis Kit (catalog no. K1622; Thermo Fisher Scientific, Waltham, MA, USA). qRT-PCR was performed using 7500 Real-Time PCR system (Applied Biosystems, Foster City, CA, USA) with Power SYBR^®^ Green PCR Master Mix (catalog no. 4309155; Applied Biosystems, Foster City, CA, USA). The integrity of the amplified DNA was confirmed by determining the melting temperature. The relative amounts of mRNA for each gene were optimized by subtracting the Ct values of each gene’ mRNA from the Ct values of β-Actin mRNA (ΔCt). The ΔCt of the control group was then subtracted from the ΔCt of the 2-O-methyl honokiol-treated groups (ΔΔCt). Expression levels were normalized to that of β-actin. Table 1 lists the specific primers for RT-PCR.

### 4.6. Western Blotting

Cells were lysed in lysis buffer containing 50 mM Tris-Cl, 150 mM NaCl, 5 mM EDTA, 0.1% NP40, and Xpert protease inhibitor cocktail Solution 100× (catalog no. p3100-010; GenDEPOT, Barker, TX, USA). Protein concentration was determined by Bradford assay. A 10–12% SDS-polyacrylamide gel was run under standard electrophoresis conditions, with 30 µg of total protein loaded in each lane. Separated proteins were transferred to Immobilon-P membranes (Millipore, Bedford, MA, USA) at 100 V for 100 min, and incubated with primary antibodies in blocking solution (3% BSA). Proteins were visualized using the Western blotting luminol reagent (catalog no. sc-2048; Santa Cruz, Dallas, TX, USA). The following antibodies were used: mouse anti-NS5B; 5B-3B1 (Enzo Life Science; Switzerland), mouse anti-NS3; sc-69938, mouse anti-NS4B; sc-52416, mouse anti-NS5A; sc-65458, goat anti-Lamin B; sc-6216, mouse anti-α-Actinin; sc-17829, mouse anti-β-actin; sc-47778, mouse anti-TRAF6; sc-8409 (Santa-Cruz, Dallas, TX, USA), rabbit anti-NF-kB(P65); 8242s, rabbit anti-p62; 8025s, and rabbit anti-LC3B; 2775s (Cell signalling, Danvers, MA, USA). All first antibody dilution was 1:1,000. Secondary antibodies used goat anti-mouse IgG; M32607, goat anti-rabbit IgG; 31460 (Invitrogen, Waltham, MA, USA), rabbit anti-goat IgG; SA007-500 (GenDEPOT, Barker, TX, USA). All secondary antibody dilution was 1:10,000.

### 4.7. Immunoprecipitation

Treated or non-treated cells were lysed in lysis buffer containing 50 mM Tris-Cl, 150 mM NaCl, 12 mM sodium deoxycholate, 1% Triton x-100, 0.1% SDS and Xpert protease inhibitor cocktail Solution 100× (catalog no. p3100-010; GenDEPOT, Barker, TX, USA), and cell lysates were cleared by centrifugation at 14,000 rpm for 15 min at 4 °C. The lysates (1 mg) were incubated with anti-TRAF6 (4 ug) for 24 h at 4 °C. Protein-A/G-conjugated agarose beads were added, followed by incubation for 4 h at 4 °C. Beads were washed three times with 1X TBST. Immunopellets were boiled with SDS-PAGE sample buffer and resolved by electrophoresis. The following antibodies were used: mouse anti-TRAF6; sc-8409, mouse anti-β-actin; sc-47778 (Santa-Cruz, Dallas, TX, USA), and rabbit anti-p62; 8025s (Cell signalling, Danvers, MA, USA).

### 4.8. Nuclear-Cytosol Fraction

Cell were grown on 100 mm tissue culture dishes and harvested with trypsin-EDTA. After mild centrifugation (1000 rpm for 5 min), pellets were then fractionated using the NE-PER Nuclear and Cytoplasmic Extraction kit (catalog no. 78833; Thermo Fisher Pierce, Rockfield, IL, USA), according to the manufacturer’s instructions. Briefly, harvested cells were washed with 1 mL ice-cold PBS, and transferred to a 1.5 mL microcentrifuge tube. Cell pellets were obtained by centrifugation at 500 × *g* for 3 min at 4 °C. The supernatant was removed, and the pellet was resuspended in 0.2 mL ice-cold CER I solution containing protease inhibitors. The suspension was vortexed for 15 s, and placed on ice for 15 min. After the addition of 11 μL of CER II solution, the suspension was vortexed for 10 s, placed on ice for 3 min, and vortexed again. The extract was centrifuged at 14,000 rpm for 5 min at 4 °C, and the supernatant, which represents the cytoplasmic fraction, was removed. The pellet, which contains the nuclei, was washed once with PBS as before, and then resuspended in 50 μL of ice-cold NER solution. The nuclei were vortexed for 15 s, and placed on ice for 10 min. This was repeated 3 times for a total of 40 min. The extract was centrifuged as before for 10 min, and the supernatant, which represents the nuclear fraction, was removed. Nuclear and cytoplasmic fractions were prepared in SDS-PAGE sample buffer and were analyzed by SDS-PAGE.

### 4.9. Confocal Immunofluorescence Microscopy

The cells were fixed with 4% formaldehyde in phosphate-buffered saline (PBS) for 15 min, permeabilized with 0.1% Triton x-100 in PBS for 15 min, and blocked with 0.1% NP-40 containing 3% bovine serum albumin (BSA) for 30 min. Immunofluorescence assays were performed using the rabbit anti-NF-kB(p65) (catalog no. 8242s; cell signalling, Danvers, MA, USA) primary antibodies in 0.1% NP-40 containing 3% BSA, dilution 1:100 for 1 h, followed by incubation with goat anti-rabbit Alexa Fluor 594-conjugated secondary antibodies from ThermoFisher Scientific (catalog no. A-11012; Thermo Fisher Scientific, Waltham, MA, USA), dilution 1:100 for 1 h. Then cells were DAPI (Sigma–Aldrich, St. Louis, MO, USA) stained (1 mg/mL) for 3 min. All images were taken with a Carl Zeiss LSM-800 confocal microscopy system. All images were analyzed with Zen Black Edition lite, version 2009.

### 4.10. IFN-α ELISA

After transfection with or without siRNA for 48 h, cells were treated with or without 50 μM 2-O-methylhonokiol for 24 or 48 h, and then washed with 1X PBS buffer. Serum-free media was added, and cells were incubated for 24 h. This supernatant was harvested for IFN-α detection using an ELISA kit. The ELISA was performed using the Human IFN-α ELISA kit (Catalog no. E-EL-H6125; Elabscience, Houston, TX, USA), according to the manufacturer’s instructions.

### 4.11. Statistical Analysis

Each experiment was performed at least five times, and the results were expressed as means ± SD. Depending on the design of the experiment, data were analyzed with Student’s *t*-tests and one-way ANOVA/post hoc multicomparison analysis (Tukey’s test). A value of *p* < 0.05 was considered to be a statistically significant difference. All statistical analysis was performed using Microsoft Excel and VassarStats site (http://vassarstats.net/, access on 18 March 2021).

## Figures and Tables

**Table 1:** List of primer couples generated for qRT-PCR.

Gene	Direction	Sequence
HCV	Forward	GTCTAGCCATGGCGTTAGTATGAG
	Reverse	CTTGTGGTAGCCTGATAGGGT
b-Actin	Forward	CATCGAGCACGGCATCGTCA
	Reverse	TCGAAGTCCAGGGCGACATA
TRAF6	Forward	TTTGCTCTTATGGATTGTCCCC
	Reverse	CATTGATGCAGCACAGTTGTC
P65	Forward	CTGTGCGTGTCTCCATGCA
	Reverse	TCGTCTGTATCTGGCAGGTACTG
IFN-α	Forward	TTTCTCCTGCCTGAAGGACAG
	Reverse	GCTCATGATTTCTGCTCTGACA
IFN-β	Forward	GAACTTTGACATCCCTGAGGAGATTAAGCAGC
	Reverse	GTTCCTTAGGATTTCCACTCTGACTATGGTCC
MxA	Forward	AACAACCTGTGCAGCCAGTA
	Reverse	AAGGGCAACTCCTGAGAGTG
PKR	Forward	TCTCTGGCGGTCTTCAGAAT
	Reverse	ACTCCCTGCTTCTGACGGTA

## Data Availability

The data that support the findings of this study are available on request from the corresponding author.

## Supplementary Materials

The following are available online at https://www.mdpi.com/article/10.3390/ijms22126499/s1.

## Abbreviations

HCV	Hepatitis C virus
NF-kB	Nuclear factor kappa-light-chain-enhancer of activated B cells
TRAF6	Tumor necrosis factor receptor (TNFR)-associated factor 6
INF	Interferon
DAA	Direct Acting Antiviral
PRRs	Pattern Recognition Receptors
RLRs	Retinoic acid-inducible gene I product (RIG-I)-like receptors
TLRs	Toll-like receptors
PAMPs	Pathogen-associated molecular patterns
LUBAC	Linear ubiquitin chain assembly complex
PKR	Protein kinase R
MxA	Myxovirus resistance protein 1
CQ	Chloroquine
