# Supplementary material for: 2-O-Methylhonokiol Suppresses HCV Replication via TRAF6-Mediated NF-kB Activation
Source: Int J Mol Sci. 2021 Jun 17;22(12):6499. doi: 10.3390/ijms22126499 (PMC8234778; doi:10.3390/ijms22126499)
Supplement: Supplementary file 1 [file ijms-22-06499-s001.zip › ijms-1251839-SI.pdf]

International Journal of
Molecular Sciences

MDPI

Supplementary Materials:

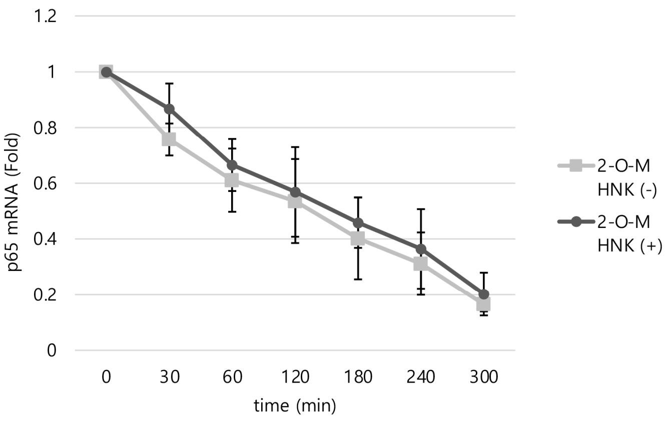

Supplementary Fig. 1 Degradation rate of 2-O-methylhonokiol-induced NF-kB(p65) mRNA after transcriptional block with actinomycin D. Huh7.5/Con1 cells were treated with 2-O-methylhonokiol for 24hr. Then actinomycin D (1ug/ml) was added, and cells were incubated for various lengths of time followed by RNA extraction. NF-kB(p65) mRNA levels were determined by real-time PCR. NF-kB(p65) mRNA levels were normalized by β-actin levels. Results are the mean±SD of three independent experiments. 2-O-M HNK, 2-O-methylhonokiol.

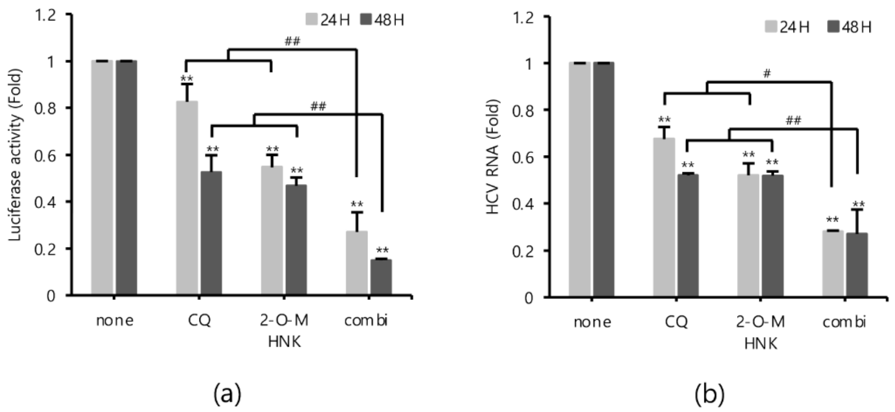

Supplementary Fig. 2 Combination effect of 2-O-methylhonokiol with chloroquine on HCV replication. Huh7.5/Con1 cells were treated with CQ 10^{-5}M, 2-O-methylhonokiol 50μM, and combination two drugs for 24h or 48h. HCV replication was measured by *Renilla* luciferase assays (a) and qRT-PCR (b). HCV RNA levels were determined by real-time PCR. CQ, chloroquine; 2-O-M HNK, 2-O-methylhonokiol. HCV RNA levels were normalized by β-actin levels. Results are the mean±SD five independent experiments. * P < 0.05, ** P <0.01 versus untreated control or versus the untreated siCon-transfected control; # P < 0.05, ## P< 0.01 versus the 2-O-methylhonokiol-treated, siCon-transfected control or versus CQ or 2-O-M HNK ; one-way ANOVA with Tukey's multiple comparisons test.
